# A Comparative Evaluation of the Dissolving Abilities of Eucalyptus, Orange, and Castor Oils in Endodontic Retreatment Using Conventional and Rotary Techniques

**DOI:** 10.7759/cureus.64063

**Published:** 2024-07-08

**Authors:** Karuna R Siraparapu, Khwaja Moinuddin, Rini Behera, Vivek Taduri, Haritha Durgam, Nimeshika Ramachandruni

**Affiliations:** 1 Conservative Dentistry and Endodontics, Malla Reddy Dental College for Women, Hyderabad, IND; 2 Department of Oral Health Sciences, Nizam’s Institute of Medical Sciences, Hyderabad, IND; 3 Conservative Dentistry and Endodontics, Institute of Dental Sciences, Bhubaneswar, IND; 4 Conservative Dentistry and Endodontics, Mamata Institute of Dental Sciences, Hyderabad, IND; 5 Dentistry, GSL Hospital, Rajahmundry, IND

**Keywords:** orange oil, natural solvents, endodontic re-treatment, eucalyptus oil, castor oil

## Abstract

Introduction: Endodontic retreatment is essential for periapical healing, involving the removal of inadequate fillings, thorough cleaning, and new filling application to prevent leakage. This study compares the dissolving abilities of Eucalyptus, Orange, and Castor oils in the re-treatment of resin-based endodontic fillings using conventional and rotary techniques.

Methodology: Thirty single-rooted human teeth were prepared and filled with gutta-percha and AH Plus sealer. They were divided into three groups (n=10) based on the solvent used (Eucalyptus, Orange, or Castor oil) and further subdivided based on the techniques used (conventional and rotary). Standardised re-treatment procedures were performed, and the amount of residual material was measured.

Results: A significant difference (p<0.001) was found among the groups, indicating that both the type of solvent and the technique significantly affected the amount of residual material. The rotary technique generally left less residual material compared to the conventional technique for all solvents. Eucalyptus oil with the rotary technique showed the least residual material (mean = 5.8), while Castor oil with the conventional technique showed the most (mean = 10.2).

Conclusion: Eucalyptus oil, especially when used with rotary techniques, is highly effective in removing resin-based endodontic fillings, providing a viable and safer alternative to traditional solvents. The study underscores the importance of selecting appropriate solvents and techniques for successful endodontic re-treatment.

## Introduction

Endodontic re-treatment is crucial for periapical healing, involving the removal of inadequate root canal fillings, thorough cleaning of root canals, and the application of new fillings to prevent leakage. Achieving a three-dimensional, void-free filling is essential to inactivate residual irritants and prevent their harmful effects. Typically, root canals are filled with gutta-percha points and a sealer. Gutta-percha occupies most of the root canal space, while the sealer fills irregular areas that mechanical instruments cannot reach. Re-treatment can be performed with or without solvents using various techniques, such as hand instruments, engine-driven reciprocating and rotating Ni-Ti instruments, ultrasonics, lasers, or heat-emitting devices that soften the gutta-percha [1].

The Reciproc system (VDW Dental, Munich, Germany), introduced in 2011, uses an engine-driven device with three files (R 25, R 40, and R 50) for reciprocating instrumentation. While rotary techniques have a longer history and extensive study, reciprocating techniques have also shown promise. Comparative studies yield mixed results, with some favouring rotary techniques and others finding no significant differences. Solvents are recommended for their ability to dissolve resin-based endodontic filling materials from hard-to-reach anatomical structures and Dentinal tubules [1]. Solvent-free re-treatment risks mechanical damage due to excessive friction and heat generation. Historically, chloroform was used as a solvent for root canal re-treatment, but its use has been banned due to carcinogenicity and cytotoxicity concerns [2]. Consequently, essential oils like clove, citronella, cottonseed, eucalyptus, lavender, orange, peppermint, pine needle, turpentine, thyme, white pine, and wintergreen oil have gained popularity as natural solvents [3].

Eucalyptus oil is bio-compatible and has antimicrobial properties, including antibacterial, anti-fungal, and antiviral effects. It effectively dissolves resin-based endodontic fillings [3,4]. Similarly, refined orange essential oil, extracted from sweet oranges, is favoured for its low toxicity to peri-apical tissues. Castor oil is another natural solvent being evaluated for its effectiveness in endodontic re-treatment. Existing literature on endodontic re-treatment emphasises various techniques and materials essential for periapical healing. However, there are significant gaps, particularly in the comparative studies of rotary and reciprocating techniques, the efficacy and safety of natural solvents, and the standardisation of methodologies. Natural solvents like eucalyptus, orange, and castor oil are emerging as alternatives to chloroform, but comprehensive research on their effectiveness and long-term impact is still limited. This study aims to compare and evaluate the dissolving abilities of three natural oils-Eucalyptus, Orange, and Castor oil-in the re-treatment of resin-based endodontic fillings using both conventional and rotary techniques.

## Materials and methods

This study was carried out with ethical approval from the S. Nialingappa Institute of Dental Sciences and Research Institutional Ethics Committee (approval no. HKES/SNIDSR/IEC/SS/10/22). The sample consisted of permanent maxillary and mandibular single-rooted human teeth with fully developed roots, ensuring they were free from decay, external resorption, and previous endodontic treatments. These teeth were extracted in the Department of Oral Surgery due to periodontal, orthodontic, or other medical reasons. After extraction, the teeth were cleaned of any soft tissue deposits, rinsed under running water, and stored in a saline solution to maintain their integrity.

Sample preparation

Access cavities were prepared in each tooth, and the pulp tissue was completely removed. The length of the root canals was determined using a K-file, ensuring precise measurement for subsequent procedures. The canals were prepared apically up to ISO size #40, with irrigation using 1 ml of 2.5% sodium hypochlorite between the use of each instrument to ensure disinfection.

To remove the smear layer, which can interfere with the bonding of the filling material, 1 ml of 2.5% sodium hypochlorite was used for 30 seconds, followed by 1 ml of 15% ethylenediaminetetraacetic acid (EDTA) for 60 seconds, and a final rinse with saline. The canals were then dried using sterile paper points to prepare for the filling process.

Root canal filling

The canals were filled with standard gutta-percha (GP) points and AH Plus sealer, utilising the cold lateral condensation technique according to the manufacturer's guidelines. This method ensures a dense, three-dimensional filling of the canal space to prevent any micro-leakage and potential reinfection.

Experimental groups

Thirty root canals were divided into three groups (n = 10) based on the solvents and instrumentation techniques used for re-treatment, with each group further subdivided into two subgroups. Groupings are as follows: group 1-Eucalyptus oil conventional hand instruments; group 2-Eucalyptus oil Rotary; group 3-orange oil conventional hand instruments; group 4-orange oil Rotary; group 5-castor oil hand instruments; and group 6-castor oil Rotary group.

Subgroup A - Manual Re-treatment With Solvent

After storage in saline for seven days, the canals in the first subgroup underwent re-treatment with a solvent. A K-reamer #15 (Mani, Tochigi, Japan) was rotated 120° clockwise with slight apical pressure to create a solvent path. The softened gutta-percha was then removed using an H-file up to size #40 (Mani, Tochigi, Japan). This process was repeated until all visible filling material was removed from the canal.

Subgroup B - Engine-driven Re-treatment With Neoendo Instruments

The second subgroup also began with solvent application. However, instead of manual files, engine-driven Neo endo re-treatment instruments (Orikam Healthcare, India) were used. The Neo endo instruments (N 1, N 2, and N 3) were sequentially applied to remove gutta-percha from the coronal to the apical thirds of the canals. This method aimed to ensure thorough and efficient cleaning of the canal spaces.

Post-treatment evaluation

**Figure 1:**
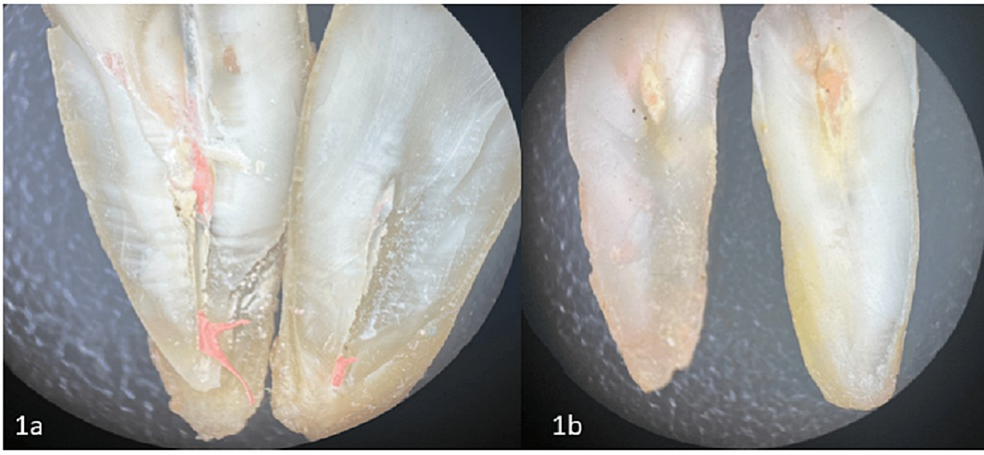
Stereo-microscopic images of Eucalyptus oil group 1a) Group 1 subgroup 1: Images of Eucalyptus oil conventional hand instruments group; 1b) Group 1 subgroup 2: Images of Eucalyptus oil Rotary group.

**Figure 2:**
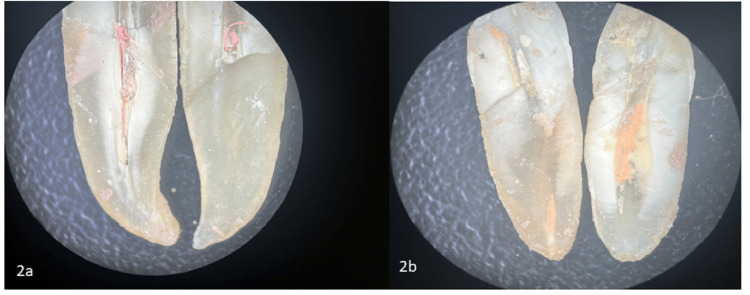
Stereo-microscopic images of orange oil group 2a) Group 2 subgroup 1: Images of orange oil conventional hand instruments group. 2b) Group 2 subgroup 2: Images of orange oil Rotary group.

**Figure 3:**
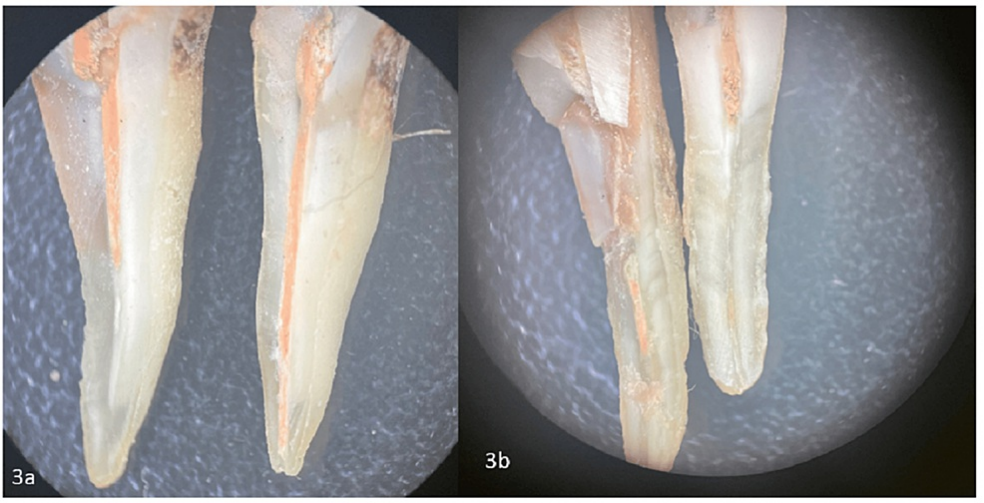
Stereo-microscopic images of castor oil group 3a) Group 3 subgroup 1: Images of Castor oil hand instruments; 3b) Group 3 subgroup 2: Castor oil Rotary group.

After the re-treatment procedures, the canals were evaluated to ensure complete removal of the old filling materials and proper cleaning. The effectiveness of each method was assessed by examining the canal walls for residual debris and unremoved gutta-percha using microscopy imaging (Figures 1-3). The results from the different groups were compared to determine the most efficient re-treatment technique.

Statistical analysis

Statistical analyses were conducted using SPSS software, version 20.0 (IBM Corp., Armonk, NY). Descriptive statistics, including mean and standard deviation (SD), were used to summarise quantitative data. Intergroup comparisons were assessed with post hoc pairwise comparisons performed using the Wilcoxon signed-rank test. A p-value of <0.05 was considered statistically significant, indicating meaningful differences throughout the study.

## Results

The study assessed the effectiveness of three natural solvents, Eucalyptus oil, Orange oil, and Castor oil, in removing resin-based endodontic filling material, comparing conventional and rotary techniques. A statistically significant difference was found among the groups, with a p-value of <0.001, indicating that both the type of solvent and the technique used significantly influenced the amount of residual material left in the root canals. The results demonstrated that the rotary technique generally left less residual material compared to the conventional technique for all solvents tested. Specifically, Eucalyptus oil used with the rotary technique showed the least residual material (mean = 5.8), while Castor oil used with the conventional technique showed the most residual material (mean = 10.2). This highlights the superior performance of Eucalyptus oil and the rotary technique in minimising residual filling material, as detailed in Table 1.

**Table 1:** Comparison of mean scores for different oils using conventional and rotary methods This table shows the comparison of mean scores (Mean ± SD) for different oils using conventional and rotary methods. 'N' represents the number of samples in each group. The p-value was calculated using ANOVA.

Treatment Group	Method	N	Mean ± SD	Std. Error	95% Confidence Interval for Mean		Minimum	Maximum
			7.200 ± 0.4472		Lower Bound	Upper Bound		
Eucalyptus oil	Conventional	5	5.800 ± 0.4472	.2000	6.645	7.755	7.0	8.0
	Rotary	5	7.800 ± 0.4472	.2000	5.245	6.355	5.0	6.0
Orange oil	Conventional	5	6.600 ± 0.2236	.2000	7.245	8.355	7.0	8.0
	Rotary	5	10.200 ± 0.4472	.1000	6.322	6.878	6.5	7.0
Castor oil	Conventional	5	8.800 ± 0.4472	.2000	9.645	10.755	10.0	11.0
	Rotary	5		.2000	8.245	9.355	8.0	9.0
P value								<0.001*

The post hoc pairwise comparison results, detailed in Table 2, indicated significant differences in mean residual material among the groups. Eucalyptus oil comparisons: Eucalyptus oil (rotary) showed significantly less residual material compared to both Orange oil (conventional and rotary) and Castor oil (conventional and rotary) (p-values < 0.05). Orange oil comparisons: Orange oil (rotary) showed significantly less residual material compared to Castor oil (conventional and rotary) (p-values < 0.05). Castor oil comparisons: Castor oil (rotary) was more effective than the conventional method, but still left more residual material compared to Eucalyptus and Orange oils.

**Table 2:** Post hoc pairwise comparison of mean differences between groups This table presents the mean differences between groups along with the standard error, p-values, and 95% confidence intervals. The p-values were derived using paired t-tests.

	Mean Difference	Std. Error	P value	95% Confidence Interval	
				Lower Bound	Upper Bound
1-2	1.4000^*^	.2646	.000*	.582	2.218
1-3	-.6000	.2646	.026*	-1.418	.218
1-4	.6000	.2646	.026*	-.218	1.418
1-5	-1.6000^*^	.2646	.000*	-2.418	-.782
1-6	-3.0000^*^	.2646	.000*	-3.818	-2.182
2-3	-2.0000^*^	.2646	.000*	-2.818	-1.182
2-4	-.8000	.2646	.050*	-1.618	.018
2-5	-3.0000^*^	.2646	.000*	-3.818	-2.182
2-6	-4.4000^*^	.2646	.000*	-5.218	-3.582
3-4	1.2000^*^	.2646	.002*	.382	2.018
3-5	-1.0000^*^	.2646	.010*	-1.818	-.182
3-6	-2.4000^*^	.2646	.000*	-3.218	-1.582
4-5	-2.2000^*^	.2646	.000*	-3.018	-1.382
4-6	-3.6000^*^	.2646	.000*	-4.418	-2.782
5-6	-1.4000^*^	.2646	.000*	-2.218	-.582

Overall, the analysis highlights the superior performance of Eucalyptus oil and the rotary technique in reducing residual endodontic filling material. The significant differences among the groups underscore the importance of selecting an appropriate solvent and technique for effective endodontic re-treatment. Eucalyptus oil, especially when used with the rotary technique, demonstrated the best results, making it a preferable choice for clinicians aiming to minimise residual filling material in root canals.

## Discussion

Endodontic failure signifies the inability of primary endodontic treatment to effectively heal and maintain the integrity of the root canal system, often resulting in persistent or recurring symptoms. This failure can be attributed to two main categories: technical and biological factors. Technical issues stem from challenges associated with the initial root canal treatment, such as inadequate filling, incomplete removal of diseased pulp tissue or dentin debris, and complexities in managing the root canal's anatomical variations. Conversely, biological causes involve ongoing disease due to bacterial survival or untreated canals [5]. Insufficient cleaning during endodontic therapy allows bacteria to persist within the root canal system, increasing the likelihood of recurrent infections and ultimately leading to treatment failure [6,7]. When dealing with persistent endodontic issues or failures, treatment options typically include either endodontic re-treatment or surgery. Endodontic surgery involves procedures like surgical removal of the root apices followed by sealing the root end to address the problem [8]. These surgical techniques, including apical surgery, crown and root resections, perforation repairs, and intentional re-plantation, aim to correct teeth with a history of unsuccessful prior treatments. Endodontic microsurgery improves upon traditional methods by utilising high-magnification tools, ultrasonic root-end preparation, and bio-compatible materials for effective root-end sealing [9]. Advanced technologies like the dental operating microscope, cone beam computed tomography for precise diagnostics, and piezoelectric devices for osteotomy and root-end preparation are integral to modern endodontic surgery [10].

Re-treatment serves as the primary recourse when initial root canal treatments exhibit inadequacies such as sensitivity to palpation, localised swelling, recurrent caries, leaky provisional restorations, or substandard coronal restorations. Radiographic evaluations often reveal untreated canals, poorly filled canals with voids, separated instruments, or recurrent caries missed during clinical examination. Ensuring thorough removal of filling materials from the root canal system is critical for the success of non-surgical endodontic re-treatment. Residual filling material within root canals can harbour bacteria or necrotic tissue, potentially causing periapical inflammation or treatment failure [11,12]. Several methods are employed to achieve gutta-percha removal. Heat carriers like Touch N. Heat or System B are commonly utilised to remove the coronal aspect of gutta-percha. Additionally, Gates-Glidden burs have proven effective in this process [13].

While nickel-titanium rotary instruments have been developed to effectively clean and shape root canals, maintain canal morphology, avoid deformation, and reduce treatment time, manual instrumentation and re-treatment remain prevalent in clinical practice due to cost and availability considerations [14,15].

Previously favoured for its rapid action and easy evaporation, chloroform's use in gutta-percha removal has declined due to concerns over potential carcinogenicity and messiness. Xylene, another solvent, offers more controlled gutta-percha softening but poses risks of neuro-toxicity and peri-apical tissue damage, necessitating cautious handling [2,16]. In response to these safety concerns, essential oils have emerged as safer alternatives [17,18]. Bio-compatible and non-carcinogenic, essential oils such as Eucalyptus oil and Sweet Orange oil possess antiseptic properties that facilitate gutta-percha dissolution. Eucalyptus oil, derived from Eucalyptus globulus leaves through steam distillation, contains 1,8-cineole, known for its anti-inflammatory and antibacterial properties. Similarly, Sweet Orange oil, extracted via cold press, contains d-limonene, effective in gutta-percha dissolution, although their precise mechanisms in dissolving endodontic sealers require further study [17-19].

This study aimed to evaluate the efficacy of three natural solvents, Eucalyptus oil, Orange oil, and Castor oil, in removing resin-based endodontic fillings using both conventional and rotary techniques. The results demonstrate significant differences in residual material among the solvents and techniques employed, highlighting the impact of solvent type and technique on efficacy. Previous studies by Karlović et al. and Zaccaro-Scelza et al. indicated comparable efficacy between essential oils and traditional solvents like chloroform and eucalyptol in gutta-percha removal [4,20].

The findings underscore that rotary techniques generally outperform conventional methods in minimising residual material, consistent with prior research emphasising benefits such as maintaining canal shape, reducing procedural errors, and saving time [15]. Particularly noteworthy was the superior performance of Eucalyptus oil with rotary instrumentation, resulting in the least residual material (mean = 5.8%). This efficacy is attributed to 1,8-cineole's potent anti-inflammatory and antibacterial properties, making Eucalyptus oil particularly suitable for effective endodontic re-treatment.

Conversely, Castor oil, particularly with conventional techniques, yielded the highest residual material (mean = 10.2%), suggesting limitations in its effectiveness compared to Eucalyptus and Orange oils, especially with manual instrumentation. This highlights the need for further research to optimise Castor oil's application in clinical settings.

The superior performance of Eucalyptus oil and rotary techniques aligns with growing evidence supporting essential oils as safer alternatives to chloroform and xylene, known for their carcinogenic and toxic risks [15,17,18]. Essential oils offer a safer profile while maintaining effective dissolution properties, supporting their adoption in modern endodontic practice.

This study found that machine re-treatment using Eucalyptus oil achieved the lowest residual filling material (5.8%), with overall removal efficiency across experimental groups ranging between 92.77% and 93.87%. These results corroborate previous findings, affirming the high effectiveness of these techniques and solvents in clinical applications [15]. Future research should continue exploring novel solvent formulations and refining techniques to enhance the safety and efficacy of endodontic re-treatment procedures.

Clinical significance and limitations of the study

The study highlights the effectiveness of natural solvents, particularly Eucalyptus oil, in endodontic re-treatment. The rotary technique, combined with Eucalyptus oil, offers a promising alternative for safer and more effective canal cleaning.

The study's limitations include a small sample size and the use of only single-rooted teeth. Further research with a larger sample size and various tooth types is recommended to validate these findings.

## Conclusions

Eucalyptus oil, especially when used with rotary techniques, is highly effective in removing resin-based endodontic fillings, providing a viable and safer alternative to traditional solvents. This study underscores the importance of selecting appropriate solvents and techniques for successful endodontic re-treatment. Future research should explore the long-term effects of these natural solvents on periapical healing and investigate their efficacy across a broader range of tooth types and clinical scenarios. Additionally, developing standardised protocols for the application of these solvents in different re-treatment techniques could enhance their practical utility in clinical settings.

## References

[1] Katunarić A, Dijanić P, Jurić Kaćunić D, Matijević J, Galić N (2022). Efficiency evaluation of various solvents in retreatment of endodontic filling in extracted teeth. Acta Stomatol Croat.

[2] Kazi FM, Asghar S, Fahim MF (2018). Dissolving efficacy of different endodontic solvents for gutta percha with varying time intervals. J Pak Dent Assoc.

[3] Yadav HK, Yadav RK, Chandra A, Thakkar RR (2016). The effectiveness of eucalyptus oil, orange oil, and xylene in dissolving different endodontic sealers. J Conserv Dent.

[4] Karlović Z, Anić I, Azinović Z, Maršan T, Miletić I, Ciglar I (1998). Endodontic retreatment with eucalyptol and chloroform solvent. Acta stomatologica Croatica: International journal of oral sciences and dental medicine.

[5] Ng YL, Mann V, Gulabivala K (2011). A prospective study of the factors affecting outcomes of nonsurgical root canal treatment: part 1: periapical health. Int Endod J.

[6] Persic Bukmir R, Paljevic E, Vidas J, Glazar I, Pezelj-Ribaric S, Brekalo Prso I (2022). Is coronal restoration a predictor of posttreatment apical periodontitis?. Eur J Dent.

[7] Prada I, Micó-Muñoz P, Giner-Lluesma T, Micó-Martínez P, Collado-Castellano N, Manzano-Saiz A (2019). Influence of microbiology on endodontic failure. Literature review. Med Oral Patol Oral Cir Bucal.

[8] Dioguardi M, Stellacci C, La Femina L (2022). Comparison of endodontic failures between nonsurgical retreatment and endodontic surgery: systematic review and meta-analysis with trial sequential analysis. Medicina (Kaunas).

[9] Setzer FC, Kratchman SI (2022). Present status and future directions: surgical endodontics. Int Endod J.

[10] Schirrmeister JF, Wrbas KT, Meyer KM, Altenburger MJ, Hellwig E (2006). Efficacy of different rotary instruments for gutta-percha removal in root canal retreatment. J Endod.

[11] Tamse A, Unger U, Metzger Z, Rosenberg M (1986). Gutta-percha solvents--a comparative study. J Endod.

[12] de Oliveira DP, Barbizam JV, Trope M, Teixeira FB (2006). Comparison between gutta-percha and resilon removal using two different techniques in endodontic retreatment. J Endod.

[13] Wong R (2004). Conventional endodontic failure and retreatment. Dent Clin North Am.

[14] Cunha TC, Matos FS, Paranhos LR, Bernardino ÍM, Moura CC (2020). Influence of glide path kinematics during endodontic treatment on the occurrence and intensity of intraoperative and postoperative pain: a systematic review of randomized clinical trials. BMC Oral Health.

[15] Somma F, Cammarota G, Plotino G, Grande NM, Pameijer CH (2008). The effectiveness of manual and mechanical instrumentation for the retreatment of three different root canal filling materials. J Endod.

[16] Kaplowitz GJ (1990). Evaluation of Gutta-percha solvents. J Endod.

[17] Martos J, Gastal MT, Sommer L, Lund RG, Del Pino FA, Osinaga PW (2006). Dissolving efficacy of organic solvents on root canal sealers. Clin Oral Investig.

[18] Bukhari SM, Rashid S, Bhat R (2023). Comparative evaluation of the effectiveness of various solvents on dissolving efficacy of gutta percha- an in vitro- study. Int Dent J Student’s Res.

[19] Shah T, Ramesh S, Sugumaran S, Choudhari S (2023). Endodontic retreatment efficacy with and without solvents: a systematic review. J Conserv Dent Endod.

[20] Scelza MF, Coil JM, Maciel AC, Oliveira LR, Scelza P (2008). Comparative SEM evaluation of three solvents used in endodontic retreatment: an ex vivo study. J Appl Oral Sci.

